# Applying Tandem Mass Spectral Libraries for Solving the Critical Assessment of Small Molecule Identification (CASMI) LC/MS Challenge 2012

**DOI:** 10.3390/metabo3020312

**Published:** 2013-04-26

**Authors:** Herbert Oberacher

**Affiliations:** Institute of Legal Medicine and Core Facility Metabolomics, Innsbruck Medical University, Muellerstrasse 44, 6020 Innsbruck, Austria; E-Mail: herbert.oberacher@i-med.ac.at; Tel.: +43-512-9003-70639; Fax: +43-512-9003-73600

**Keywords:** tandem mass spectral library, identification, library search, MassBank, METLIN, NIST 12, Wiley Registry of Tandem Mass Spectral Data, MS for ID

## Abstract

The “Critical Assessment of Small Molecule Identification” (CASMI) contest was aimed in testing strategies for small molecule identification that are currently available in the experimental and computational mass spectrometry community. We have applied tandem mass spectral library search to solve Category 2 of the CASMI Challenge 2012 (best identification for high resolution LC/MS data). More than 230,000 tandem mass spectra part of four well established libraries (MassBank, the collection of tandem mass spectra of the “NIST/NIH/EPA Mass Spectral Library 2012”, METLIN, and the ‘Wiley Registry of Tandem Mass Spectral Data, MSforID’) were searched. The sample spectra acquired in positive ion mode were processed. Seven out of 12 challenges did not produce putative positive matches, simply because reference spectra were not available for the compounds searched. This suggests that to some extent the limited coverage of chemical space with high-quality reference spectra is still a problem encountered in tandem mass spectral library search. Solutions were submitted for five challenges. Three compounds were correctly identified (kanamycin A, benzyldiphenylphosphine oxide, and 1-isopropyl-5-methyl-1H-indole-2,3-dione). In the absence of any reference spectrum, a false positive identification was obtained for 1-aminoanthraquinone by matching the corresponding sample spectrum to the structurally related compounds N-phenylphthalimide and 2-aminoanthraquinone. Another false positive result was submitted for 1H-benz[g]indole; for the 1H-benz[g]indole-specific sample spectra provided, carbazole was listed as the best matching compound. In this case, the quality of the available 1H-benz[g]indole-specific reference spectra was found to hamper unequivocal identification.

## 1. Introduction

Tandem mass spectral libraries are valuable resources of mass spectral information [1] and a number of libraries have been developed for the identification of small molecules, including metabolites, drugs and toxins [2,3,4,5,6,7,8,9,10,11,12,13]. State-of-the-art libraries are considered to allow sensitive, specific and robust compound identification [6,14,15,16]. Typically, advanced library structures (e.g., comprehensive coverage of compound-specific breakdown curves with reference spectra acquired at different collision energy (CE) settings) and tailor-made search algorithms are combined to create efficient tools for compound identification.

The “Critical Assessment of Small Molecule Identification” (CASMI) contest was aimed in testing strategies for small molecule identification that are currently available in the experimental and computational mass spectrometry community. In CASMI spectral information for a set of compounds was published, and the participants were asked to determine either the molecular formula or the molecular structure. We participated in Category 2 of the CASMI Challenge 2012 (best identification for high resolution LC/MS data) and applied four well established tandem mass spectral libraries for identifying the unknown compounds. The libraries used included MassBank [9], the collection of tandem mass spectra part of the “NIST/NIH/EPA Mass Spectral Library” (NIST MS/MS 2012) [17], METLIN [3], and the ‘Wiley Registry of Tandem Mass Spectral Data, MSforID’ (Wiley Registry MS/MS) [5,6,7,18]. Each library was searched with the accompanied search algorithm.

MassBank is a public repository of mass spectra of small chemical compounds [9]. Research groups contributing to the repository make their mass spectral data available to the public as supporting experimental data for other researchers. MassBank contains tandem mass spectra acquired on a variety of mass spectrometers, including diverse low- and high-resolution instruments. Users of MassBank are provided with informatics tools to search the distributed data for identification of chemical compounds detected by mass spectrometry (MS) and tandem mass spectrometry (MS/MS).

The NIST MS/MS 2012 database is part of the NIST/NIH/EPA Mass Spectral Library [17]. The library covers small (bio-)organic molecules and peptides. Reference spectra were acquired on different types of mass spectrometers, including diverse low- and high-resolution instruments. The NIST MS/MS 2012 database comes bundled with a search algorithm optimized for tandem mass spectral library search (NIST MS Search program 2.0 g).

METLIN, a freely accessible web-based data repository, has been developed to assist in a broad array of metabolite research and to facilitate metabolite identification through mass analysis [3,19]. METLIN contains resources for characterizing known and unknown metabolites, including a database of high-resolution tandem mass spectra. The tandem mass spectral library was developed on a quadrupole-quadrupole-time-of-flight instrument (QqTOF) by collecting compound-specific reference spectra at four different collision energies (CE).

The Wiley Registry MS/MS is a tandem mass spectral library allowing sensitive, specific, and robust identification of small (bio-)organic molecules [5,6,7,18]. The library was developed on a QqTOF instrument employing 10 different collision energies for fragmentation. The library comes bundled with a tailor-made search algorithm (MSforID Search). The Wiley Registry MS/MS was extensively tested [6,7,14,15,16]. The results of multicentre and cross-validation studies clearly suggest that the Wiley Registry MS/MS allows reliable and robust identification with data acquired on various instruments. The proven transferability is a unique characteristic distinguishing the Wiley Registry MS/MS from all other tandem mass spectral libraries currently available. The current version of the Wiley Registry MS/MS was successfully applied in forensic applications to the identification of drugs and metabolites thereof [5,20,21].

By using four tandem mass spectral libraries, more than 230,000 reference spectra were available for solving the CASMI challenges. Although it was expected that only a portion of the chemical space would be covered by the available libraries, a considerable number of putative positive identifications were obtained. Herein, a detailed summary of the library search results is provided. Furthermore, the cause of false positive results is discussed.

## 2. Results and Discussion

For solving the CASMI LC/MS Challenge 2012 four different tandem mass spectral libraries were searched with the mass spectral data provided. The libraries selected included MassBank, the NIST MS/MS 2012 database, METLIN, and the Wiley Registry MS/MS. Each library was searched with the accompanied search algorithm. The data acquired in positive ion mode was processed. For five out of 12 challenges solutions were submitted. The putative positive identifications are summarized in Table 1. Three compounds were correctly identified (kanamycin A, benzyldiphenylphosphine oxide, and 1-isopropyl-5-methyl-1H-indole-2,3-dione). The spectrum of 1-aminoanthraquinone was matched to the structurally related compounds N-phenylphthalimide and 2-aminoanthraquinone. In this case a false positive result was created due to the absence of reference spectra either in the Wiley Registry MS/MS or any other library selected. Another false positive result was submitted for 1H-benz[g]indole; for the provided sample spectra, MassBank listed carbazole as the best matching compound.

**Table 1:** Putative identifications submitted.

Challenge	Matched compound(s)	Library	Score	Correct compound
1	Kanamycin A	MassBank, NIST MS/MS 2012, Wiley Registry MS/MS	1.0	Kanamycin A
10	N-Phenylphthalimide	Wiley Registry	1.0	1-Aminoanthraquinone
	2-Aminoanthraquinone	MS/MS	0.84	
13	Benzyldiphenylphosphine oxide	MassBank	1.0	Benzyldiphenylphosphine oxide
14	Carbazole	MassBank	1.0	1H-Benz[g]indole
	1H-benz[g]indole		0.99	
15	1-Isopropyl-5-methyl-1H-indole-2,3-dione	MassBank	1.0	1-Isopropyl-5-methyl-1H-indole-2,3-dione

After the official release of the correct solutions, the content of all libraries was checked for the availability of reference spectra representing the seven unidentified compounds. Such spectra would have indicated the occurrence of false negative results. This was, however, excluded because the compounds were not present in the tandem mass spectral libraries used.

The majority of compounds were identified with MassBank. This result, however, should be interpreted with caution; the number of matches is not a sign of superior performance. For three out of four compounds identified with MassBank (benzyldiphenylphosphine oxide, 1H-benz[g]indole, 1-isopropyl-5-methyl-1H-indole-2,3-dione), reference spectra were exclusively found in this library. Kanamycin A was the only compound with reference spectra available in two different libraries, and these libraries provided correct positive matches.

Tandem mass spectral library search is a sensitive and specific tool for small molecular identification. Depending on the field of research, however, the number of knowns included in available libraries is often low, and the number of compounds represented by high-quality reference spectra is even lower. Due to the increasing acceptance and need in qualitative analysis, enlargement of libraries is gaining more and more importance. Tandem mass spectra can be created on diverse instruments employing a range of instrumental settings. The ultimate tandem mass spectral library would contain compound-specific spectra created with any experimental setup principally available. There is evidence that a reliable, transferable and robust tandem mass spectral library can be created even by using a single instrument [6,7,14,15,16]. Based on this observation, it should become possible to define minimal standards for acquiring representative reference spectra to facilitate transfer, sharing, and merging of libraries.

The CASMI contest seems to provide valuable information on the principle usefulness of tandem mass spectral library search for small molecular identification. It is important to note, however, that the number of sample spectra was too low to expect any clear answer in relation to the question of performance differences between tandem mass spectral library search packages (libraries and the accompanied search algorithms). A much higher number of positive controls and negative controls would have been needed to rate performance with the statistical parameters sensitivity and specificity, and ideally these spectra should have been acquired on diverse low- and high-resolution instruments.

### 2.1. MassBank Results

**Figure 1:**
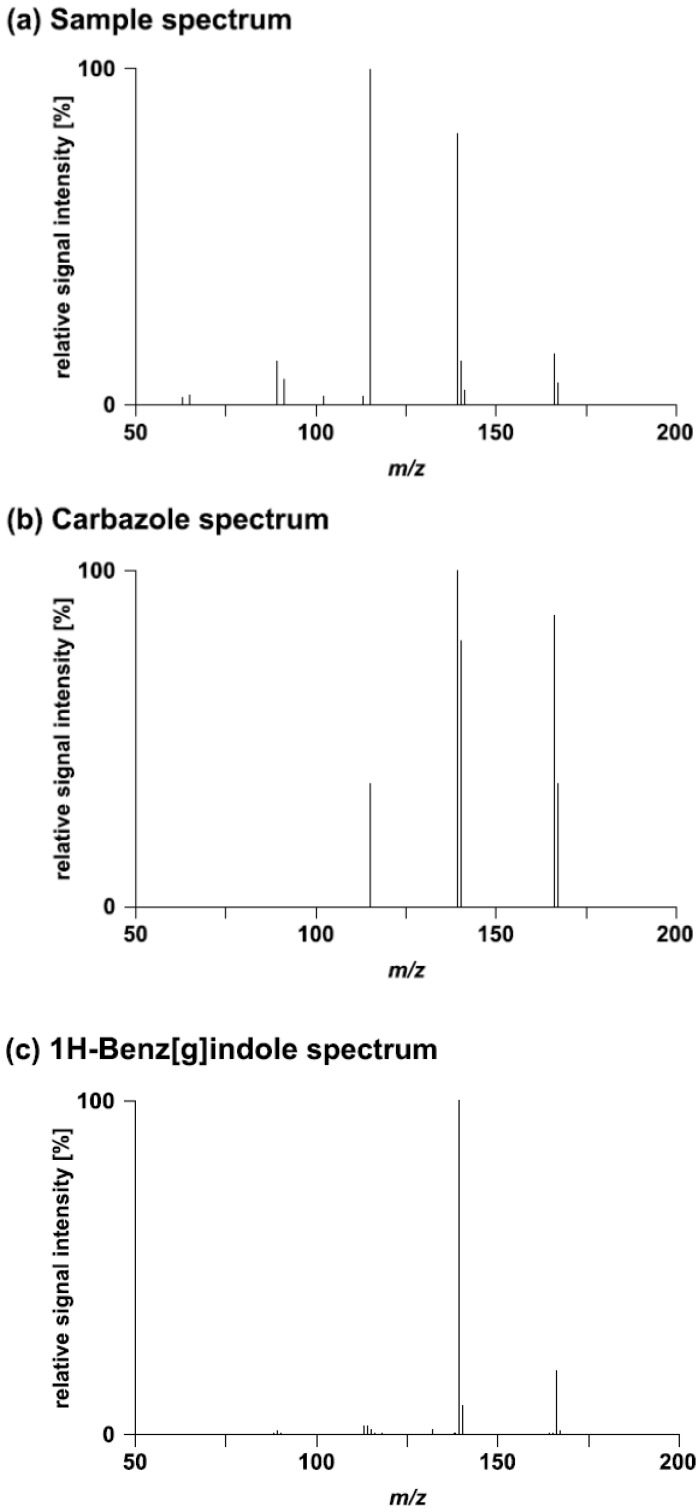
Comparison of (**a**) the HCD 180 sample spectrum of Challenge 14 with (**b**) the spectrum of the best matching compound and (**c**) the spectrum of the correct compound. The two reference spectra (**b,c**) were extracted from MassBank.

A full summary of the library search results with MassBank is provided in supplementary Table S1. For four challenges putative positive hits were obtained (Table 2). The solutions provided for Challenges 1, 13, and 15 were found to be correct. The sample spectra provided for Challenge 13 were directly extracted from MassBank. Carbazole (Challenge 14) was a false positive match even though a high degree of similarity was found between the sample and the reference spectra (Figure 1). The correct solution was 1H-benz[g]indole. For the higher energy collision dissociation (HCD) 180 spectrum, this compound was ranked on position 3 of the hit list (Score: 0.80).

**Table 2:** Putative identifications with MassBank. Unless otherwise stated, information on the best matching compound only is provided.

Challenge	CE	Matched compound(s)	Accession	Score
1	10 eV	Kanamycin A (2nd place in hit list)	KO009014	0.733
	20 eV	Kanamycin A	KO009015	0.874
	30 eV	Kanamycin A (2nd place in hit list)	KO009015	0.773
13	25%	Benzyldiphenylphosphine oxide	UF011001	0.986
	45%	Benzyldiphenylphosphine oxide	UF011003	0.995
	HCD 45	Benzyldiphenylphosphine oxide	UF011007	0.996
	HCD 75	Benzyldiphenylphosphine oxide	UF011009	0.967
14	HCD 120	Carbazole (2nd place in hit list)	UF026312	0.807
		1H-Benz[g]indole (7th place in hit list)	UF011409	0.725
	HCD 180	Carbazole	UF026314	0.819
		1H-Benz[g]indole (3rd place in hit list)	UF011413	0.801
15	HCD 120	1-Isopropyl-5-methyl-1H-indole-2,3-dione	UF011512	0.968
	HCD 90	1-Isopropyl-5-methyl-1H-indole-2,3-dione	UF011510	0.971

### 2.2. NIST MS/MS 2012 Results

A full summary of the library search results with the NIST MS/MS 2012 library is provided in supplementary Table S2. For Challenge 1, kanamycin A was retrieved as correct positive match (Table 3).

**Table 3:** Putative identifications with the NIST MS/MS 2012 database. Information on the best matching compound only is provided.

Challenge	CE	Matched compound	MF	RMF	Prob
1	10 eV	Kanamycin A	645	879	98.88
	20 eV	Kanamycin A	501	833	98.81
	30 eV	Kanamycin A	531	776	98.66

### 2.3. METLIN Results

A full summary of the library search results obtained with METLIN is provided in supplementary Table S3. None of the challenges were solved by searching METLIN.

### 2.4. Wiley Registry MS/MS Results

#### 2.4.1. Search Results with the Published Library

A full summary of the library search results with the Wiley Registry MS/MS is provided in supplementary Table S4. For Challenge 10 two putative positive hits were obtained (Table 4). The challenge data matched to N-phenylphthalimide and 2-aminoanthraquinone. The correct solution was 1-aminoanthraquinone, but this compound was not included in the library. Challenge 1 spectra matched tobramycin with high *ramp*-values (Table 4). The *m/z* of tobramycin, however, was 16.9788 smaller than the *m/z* of the precursor ion of the searched compound. Thus, tobramycin was excluded to be the correct compound. Due to the high *ramp*-values obtained, however, a compound with high structurally similarity to tobramycin was considered to represent the correct compound [22]. Within PubChem Compound [23] kanamycin was identified as that related compound.

**Table 4:** Putative identifications with the Wiley Registry MS/MS. Unless otherwise stated, information on the best matching compound only is provided.

Challenge	CE	Matched compound(s)	*ramp*	*m/z*	Delta
1	10 eV	Tobramycin	72.03	468.2665	16.9788
	20 eV	Tobramycin	68.43	468.2665	16.9788
	30 eV	Tobramycin	65.85	468.2665	16.9788
10	35%	N-Phenylphthalimide (1st place in hit list)	54.1	224.0707	0.0004
		2-Aminoanthraquinone (2nd place in hit list)	45.6	224.0707	0.0004

#### 2.4.1. Search Results with the Extended Library

After the official release of the correct solutions, we tried to check experimentally some of the initial results obtained. Therefore, an extended version of the Wiley Registry MS/MS was created by adding spectra of kanamycin A, 1-aminoanthraquinone, benzyldiphosphine oxide, 1H-benz[g]indole, and carbazole, and the sample spectra of challenges 1, 10, 13, and 14 were matched to this new library. The results obtained are summarized in Table 5.

**Table 5:** Putative identifications with the extended Wiley Registry MS/MS. Unless otherwise stated, information on the best matching compound only is provided.

Challenge	CE	Matched compound(s)	*ramp*	*m/z*	Delta
1	10 eV	Kanamycin A	98.84	485.2454	-0.0001
	20 eV	Kanamycin A	98.46	485.2454	-0.0001
	30 eV	Kanamycin A	98.46	485.2454	-0.0001
10	35%	N-Phenylphthalimide (1st place in hit list)	36.05	224.0707	0.0004
		1-Aminoanthraquinone (2nd place in hit list)	33.37	224.0707	0.0004
		2-Aminoanthraquinone (3rd place in hit list)	30.39	224.0707	0.0004
13	25%	Benzyldiphenylphosphine oxide	81.58	293.1090	0.0007
	45%	Benzyldiphenylphosphine oxide	91.53	293.1090	0.0007
	HCD 45	Benzyldiphenylphosphine oxide	84.05	293.1090	0.0007
	HCD 75	Benzyldiphenylphosphine oxide	83.25	293.1090	0.0007
14	HCD 120	1H-Benz[g]indole (1st place in hit list)	80.30	168.0808	-0.0004
		Carbazole (2nd place in hit list)	18.86	168.0808	-0.0004
	HCD 180	1H-Benz[g]indole (1^st^ place in hit list)	67.04	168.0808	-0.0004
		Carbazole (2nd place in hit list)	30.91	168.0808	-0.0004

For Challenge 1 kanamycin A was obtained as correct positive match by adding the corresponding reference spectra to the Wiley Registry MS/MS. Obviously, if kanamycin A spectra had been available in the published version of the Wiley Registry MS/MS, this compound would have been unequivocally identified.

The sample spectra of benzyldiphosphine oxide (Challenge 13) were found to be identical with the spectra stored in MassBank. Accordingly, we decided to verify the initial identification with an independent set of reference spectra. As expected, the compound was unequivocally identified with the extended version of the Wiley Registry MS/MS containing reference spectra of this compound.

**Figure 2:**
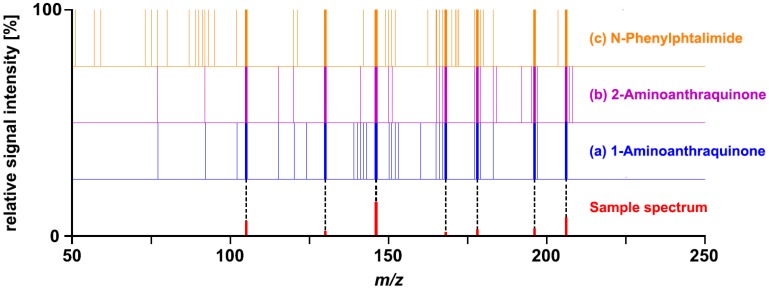
Comparison of the sample spectrum provided for Challenge 10 with the merged spectra of (**a**) 1-aminoanthraquinone, (**b**) 2-aminoanthraquinone, and (**c**) N-phenylphthalimide. Each line in the merged spectra represents a fragment ion; the corresponding signal intensities are not provided.

N-Phenylphtalimide and 2-aminoanthraquinone were obtained as hits by matching the sample spectrum of Challenge 10 to the published Wiley Registry MS/MS. It was anticipated that the addition of 1-aminoanthraquinone spectra would enable unequivocal identification. The library search result obtained with the extended version of the Wiley Registry MS/MS, however, was still inconclusive. The sample spectrum was matched with almost identical *ramp*-values to 1-aminoanthraquinone, N-phenylphtalimide, and 2-aminoanthraquinone. This observation suggests that the mass spectral information provided in Challenge 10 does not allow unequivocal differentiation of the three top-matching compounds. A visual comparison of the sample spectrum with the corresponding merged reference spectra further supported this hypothesis (Figure 2). None of the fragment ions part of the sample spectrum represented a unique identifier for 1-aminoanthraquinone. The seven fragment ions were common to all three compounds matched.

With MassBank, carbazole was the best matching solution for Challenge 14. The correct compound, however, was 1H-benz[g]indole. To check the ability of the Wiley Registry MS/MS to match the provided sample spectra to the correct compound, reference spectra of 1H-benz[g]indole and carbazole were added to the library. With the extended library, both sample spectra yielded 1H-benz[g]indole as best matching compound; carbazole was ranked second with significant lower *ramp*-values. Obviously, the sample spectra represented specific identifiers for 1H-benz[g]indole. Thus, the absence of specific reference spectra might explain the inability of MassBank to distinguish ^1^H-benz[g]indole and carbazole.

## 3. Experimental Section

The challenge data was downloaded from the contest homepage [24]. The MS/MS data acquired in positive ion mode was processed. In each case, the *m/z*-value of the precursor ion and the corresponding fragment ion mass spectrum were used as input data for library search. For Challenge 16 spectra, the *m/z* of the intact molecule and the *m/z* of the in source fragment ions were both used as precursor ion *m/z*-values. The spectra were matched to four libraries employing the accompanied search algorithms. Within the hit lists obtained, the first five matches were considered as putative positive hits. Nevertheless, only hits passing the two final examination steps were submitted. In the first control examination all matches were checked visually by direct comparison of the input spectra and the matched spectra to reject false positive hits. In the second control examination, the *m/z* of the precursor ion was used as criterion to identify false positive hits.

### 3.1. MassBank

MassBank was accessed on January 7^th^ 2013. At that time MassBank contained more than 39,000 spectra acquired on different types of instruments, including diverse low- and high-resolution instruments. Spectrum search was used to match the sample spectra to the library [25]. The search program returns a hit list of matched chemical compounds including a measure of spectral similarity. The similarity score (Score) is calculated by a weighted cosine correlation in which weighting exponents on peak intensity and the *m/z* are optimized to the MS/MS data. Spectrum search was accomplished using 0.3 units tolerance of *m/z* as well as a cutoff threshold of 5. All reference spectra collected on instruments employing atmospheric pressure ionization were allowed to match.

### 3.2. NIST MS/MS 2012

The NIST MS/MS 2012 database used in this study was published in May 2012 and contained 123,781 spectra representing 15,517 precursor ions and 7,020 compounds. Spectra were acquired on different types of instruments, including diverse low- and high-resolution instruments. A detailed description of the library can be found elsewhere [17]. The NIST MS Search program 2.0 g (NIST, Gaithersburg, MD, USA) was used for automated library search. The search program returns a hit list of matched chemical compounds including several measures of spectral similarity [26]. The Match Factor (MF) is the normalized dot product with square-root scaling of the submitted mass spectrum and a library mass spectrum, using all the elements in the submitted mass spectrum. Reverse Match Factor (RMF) is the normalized dot product with square-root scaling of the submitted mass spectrum and the library mass spectrum, but the elements that are not present in the library mass spectrum are not included. Probability (Prob) is the estimated relative likelihood of that the compound mass spectrum is the correct match for the submitted mass spectrum. Identity search was performed as “MS/MS search” using default settings. The *m/z* tolerance was set to ± 1.6 for precursor ions and ± 0.8 for product ions. Furthermore, the “ignore precursor ion” option was used.

### 3.3. METLIN

METLIN was accessed on January 9^th^ 2013. At that time METLIN contained over 55,000 high resolution MS/MS spectra obtained on a 6510 Q-TOF (Agilent Technologies, Santa Clara, CA, USA) operated in positive and negative electrospray ionization mode using four different CE (0, 10, 20 and 40V). MS/MS spectrum search was used to match the sample spectra to the library [27]. The search program returns a hit list of matched chemical compounds including a measure of spectral similarity (Metlin Score). Spectrum search was accomplished using 0.01 Da tolerance MS/MS (0.1 Da for Challenge 17) and 100 ppm tolerance of precursor. The search was restricted to [M+H]^+^ and positive ion mode. 

### 3.4. Wiley Registry MS/MS

The Wiley Registry MS/MS was developed on a QqTOF instrument (QStar XL, AB Sciex, Foster City, CA, USA) using electrospray ionization in positive and negative ion mode and 10 different collision energies for fragmentation [18]. A detailed description of the instrumental parameters applied can be found elsewhere [5,20]. At the current stage of development the library contains 12,122 spectra of 1,208 compounds. A summary of the library entries is provided elsewhere [28]. For solving the challenges, the collection of spectra acquired in positive ion mode (10,712 tandem mass spectra of 1,040 compounds) was used as reference library. An extended version of the Wiley Registry MS/MS was created by adding spectra of kanamycin A, 1-aminoanthraquinone, benzyldiphosphine oxide, 1H-benz[g]indole, and carbazole. The compounds were obtained from Sigma-Aldrich (St. Louis, MO, USA). MSforID Search [5,7] was accomplished with a program written in Pascal using Delphi 6 for Windows (Borland Software Corporation, Scotts Valley, CA, USA; now Embarcadero Technologies, Inc., San Francisco, CA, USA) using the following search parameters: *m/z* tolerance of ± 0.01 (± 0.1 for Challenge 17), intensity cut-off factor of 0.05. The search program returns a hit list of matched chemical compounds including a measure of spectral similarity (*ramp*).

## 4. Conclusions

We have applied tandem mass spectral library search to solve the CASMI LC/MS Challenge 2012. We have processed 12 challenges of category 2 representing data acquired in positive ion mode. Although more than 230,000 reference spectra were searched, putative positive results were only submitted for five challenges. Correct positive identifications were obtained in three cases. Two false positive identifications were caused by the limited specificity of either the sample spectrum provided or the reference spectra available. Despite considerable success of the library search approach particularly in comparison to *de novo* identification tools, the limited coverage of the chemical space with high- quality reference spectra still represents a problem encountered with tandem mass spectral library search. Currently, there is much effort put in the development and extension of tandem mass spectral libraries. Particularly those libraries that implement spectra from diverse resources, including low-and high-resolution instruments exhibit large growth rates. There are, however, doubts on the usefulness of such an approach for the creation of a reliable, robust and transferable identification tool [29]. Thus, in future either CASMI or similar contests should be used to evaluate and compare the performance of available tandem mass spectral libraries to identify the most appropriate strategy for library development in terms of instrumentation and CE settings. Such efforts could culminate in recommendations for a unified library design, which in due consequence would reduce redundancies, would avoid parallel developments, and would increase the overall growth rate of reliable, unique, and transferable tandem mass spectral data. In this way tandem mass spectral libraries will significantly gain credibility, which is of utmost importance to attract potential users. Furthermore, the availability of large amounts of high-quality data will stimulate the development of advanced algorithms and software tools for the efficient and reliable structure elucidation of unknown compounds.

## Supplementary Files

Supplementary File 1 Supplementary (ZIP, 21 KB)
